# Healthcare provider approaches to managing the health harms of xylazine: A qualitative study

**DOI:** 10.1016/j.josat.2026.210051

**Published:** 2026-06-11

**Authors:** Patrick J.A. Kelly, Amelia Bailey, Josiah D. Rich, Katie B. Biello, Brandon D.L. Marshall, Elizabeth S. Chen, Jaclyn M.W. Hughto

**Affiliations:** a Department of Behavioral and Social Sciences, Brown University School of Public Health, 121 South Main Street, Providence, RI, 02903, USA; b Center for Health Promotion and Health Equity, Brown University School of Public Health, 121 South Main Street, Providence, RI, 02903, USA; c Center for Alcohol and Addiction Studies, Brown University School of Public Health, Providence, RI, 02903, USA; d Center of Biomedical Research Excellence on Opioids and Overdose, Rhode Island Hospital, 1125 North Main Street, Providence, RI, 02903, USA; e The Warren Alpert School of Medicine of Brown University, 222 Richmond Street, Providence, RI, 02912, USA; f Department of Epidemiology, Brown University School of Public Health, 121 South Main Street, Providence, RI, 02903, USA; g Center for Epidemiologic Research, Brown University School of Public Health, 121 South Main Street, Providence, RI, 02903, USA; h The Fenway Institute, Fenway Health, 1340 Boylston St, Boston, MA, 02215, USA; i Department of Health Services Policy and Practice, Brown University School of Public Health, 121 South Main Street, Providence, RI, 02903, USA; j Brown Center for Biomedical Informatics, Brown University Division of Biology and Medicine, 300 Richmond Street, Providence, RI, 02912, USA

**Keywords:** Fentanyl, Overdose, Qualitative, Skin wound, Treatment, Withdrawal, Xylazine, Medetomidine

## Abstract

**Introduction:**

Xylazine-adulterated fentanyl use is associated with manifold negative health outcomes, including skin and soft tissue infections, intensified opioid withdrawal, and can complicate medication for opioid use disorder (MOUD) induction. This qualitative research aimed to explore how healthcare providers test for and treat xylazine-related health harms among people who use drugs (PWUD).

**Methods:**

From February–April 2025, twelve healthcare providers with expertise in managing the health harms of xylazine from three Northeastern states completed an approximately 45-min semi-structured interview to explore organizational capacity for xylazine toxicology screening and clinical management of xylazine-associated health harms. Rapid qualitative analysis was used to analyze transcripts and produce themes.

**Results:**

Participants worked in academic institutions (*n* = 7), free-standing clinics (*n* = 5), harm reduction organizations (*n* = 4), substance use treatment programs (n = 4), hospitals (*n* = 2), and a federally qualified health center (*n* = 1). Providers infer xylazine exposure based on clinical presentation and rarely order toxicology, which is often unavailable at some institutions. Providers noted three core clinical effects of xylazine use including overdose, xylazine-associated skin wounds, and withdrawal. Respiratory support and monitoring are essential when responding to xylazine-involved overdose. Additionally, wound chronicity, limited treatment guidelines, homelessness, and stigma complicate xylazine-associated skin wound healing. Withdrawal from xylazine typically co-occurs with opioid withdrawal and is worse than opioid-only withdrawal. Xylazine may be worsening experiences of precipitated withdrawal when inducting patients onto buprenorphine. Some physicians noted that medetomidine, a sedative more potent than xylazine, is associated with withdrawal worse than xylazine withdrawal, weakening the efficacy of recently developed xylazine withdrawal protocols and underscoring the volatility of the drug supply.

**Conclusion:**

The clinical effects of xylazine do not occur in isolation from the effects of other substances and often compound one another. Findings demonstrate the complexities of care coordination and management of xylazine-associated health harms. The high level of care coordination needed for xylazine-exposed PWUD is a result of the volatile drug supply, which is likely to become increasingly harmful as new sedative type adulterants, like medetomidine, emerge.

## 1. Introduction

The United States (US) is amid the ‘fourth wave’ of the overdose crisis characterized by a rise in overdose mortality involving multiple substances, predominantly stimulants and fentanyl (Jones et al., 2020). Polysubstance overdose deaths are partially driven by a rise in and constant fluctuation of adulterants in the drug supply. Xylazine, an alpha-2 adrenergic agonist used in equine medicine that is not approved for human use, is one adulterant of concern. First utilized as an adulterant in the Puerto Rican unregulated opioid supply in the early 2000s, xylazine’s prevalence in the US unregulated fentanyl supply grew exponentially in the 2020s (Friedman et al., 2022). The Drug Enforcement Agency seized fentanyl containing xylazine in 48 states in 2022 (Drug Enforcement Agency, n.d.), indicating the widespread xylazine infiltration of the US drug supply. Xylazine is especially prevalent in mid-Atlantic and Northeast Corridor drug markets (Friedman et al., 2022). In 2021, most street fentanyl in Maryland (80%) and Philadelphia (90%) contained xylazine (Philadelphia Department of Public Health, 2022; Russell, 2023). In the years that followed, xylazine has been detected alongside fentanyl in New England drug markets at various concentrations. In Rhode Island, surveillance data collected May 2022 through January 2023 identified xylazine in 42% of 125 community-obtained drug samples (Collins et al., 2023). Other data collected January to May 2023 identified xylazine in 17% of 59 community-obtained opioid samples (Ujeneza et al., 2025).

Xylazine has worsened drug-related harms and is associated with overdose risk, skin wound development, and withdrawal (Zagorski et al., 2023). As a non-opioid sedative, xylazine suppresses consciousness (Zagorski et al., 2023), making the co-use of fentanyl especially life-threatening; indeed, nearly all xylazine-involved US-based overdoses from 2015 to 2020 co-occurred with fentanyl (Friedman et al., 2022). Naloxone, an opioid antagonist, does not directly reverse xylazine’s sedative effect, though it is antagonistic against fentanyl and remains the optimal opioid antidote (Datta et al., 2025). Given that xylazine contributes to respiratory depression that is not reversible with naloxone, there is an urgent need to understand how clinicians respond to suspected xylazine-involved overdose.

Xylazine amplifies the risk of skin wound development among those using fentanyl. Case studies suggest xylazine-associated skin wounds can be serious and appear across modes of drug use (e.g., injection, smoking) (Dowton et al., 2023; Malayala et al., 2022; Soderquist et al., 2023; Warp et al., 2024; Wei et al., 2023; Zabel, 2024). These wounds are distinct from abscesses and can progress into large wounds with eschar if untreated (Sue & Hawk, 2024; Zagorski et al., 2023). Xylazine has a vasoconstrictive effect which reduces skin perfusion and is believed to be the pathophysiology of xylazine-associated wounds (Papudesi et al., 2025). Initial evidence indicates that the effects of these wounds are profound and can lead to the development of painful skin and soft-tissue infections that may result in amputation and mobility impairment (Sue & Hawk, 2024). People with xylazine-associated skin wounds are also concerned about limb loss and appearance impacts (Kelly et al., 2025). Although studies are beginning to describe xylazine-associated skin wounds (Jones et al., 2024), evidence on their presentation and optimal management remains limited. Thus, clearer characterization of treatment approaches is needed to reduce related morbidity and mortality.

Clinical evidence of xylazine withdrawal presentation in humans is limited (National Institute on Drug Abuse, 2023; Papudesi et al., 2025). Medications for opioid use disorder (MOUD) may be efficacious at managing xylazine withdrawal. However, some xylazine-exposed PWUD have had difficulty initiating some MOUD, specifically buprenorphine-naloxone formulations, reporting that the medication did not treat withdrawal (Reed et al., 2024). Exploring how MOUD induction protocols have been adapted to preserve efficacy is foundational to establishing evidence-based induction guidelines in the era of xylazine.

The absence of a widely available point-of-care test for xylazine could complicate diagnosis and limit understanding of which treatment approaches are most effective at managing xylazine’s effects. Qualitative inquiry that explores how physicians and other healthcare providers identify, characterize, and treat xylazine exposure among PWUD is critically important but has been overlooked. As such, the purpose of this qualitative study is to articulate themes of healthcare providers’ approaches to testing for and managing xylazine exposure in the Northeastern US, a xylazine epicenter where expertise in the clinical management of xylazine originated. Findings from this exploratory study have the potential to advance clinical consensus on xylazine management in drug markets where xylazine is growing in prevalence.

## 2. Methods

### 2.1. Participants and procedures

Between February and April 2025, a convenience sample of 12 healthcare providers in Rhode Island (*n* = 7), Massachusetts (*n* = 2), and Pennsylvania (*n* = 3) participated in a semi-structured interview that explored xylazine treatment practices. To recruit participants, the first author, a male doctoral candidate in substance use research, sent 22 healthcare providers working in primary care, addiction medicine, and emergency medicine a recruitment email; the corresponding authors of xylazine-focused literature were also contacted via email. Nine people did not respond or declined due to time constraints. The first author screened twelve interested people to determine eligibility. Eligible participants were: (1) a practicing and licensed healthcare provider; (2) self-reported expertise providing care to PWUD exposed to xylazine; (3) at least 18 years old; (4) spoke and read English; and (5) willing and able to provide informed consent. Interested and eligible participants provided verbal consent, answered demographic questions, and completed an approximately 45-min, one-on-one, audio-recorded virtual semi-structured interview with the first author. After each interview, the interviewer wrote analytic memos documenting emerging concepts. No new thematic content appeared after the tenth interview, indicating thematic saturation consistent with qualitative methodological guidance (Hennink et al., 2017); two additional interviews were conducted beyond saturation because participants were already scheduled. Participants received a $50.00 Amazon e-gift card. The Brown University Institutional Review Board approved this research. This article follows the Consolidated Criteria for Reporting Qualitative Research (COREQ) reporting checklist (Tong et al., 2007).

#### 2.1.1. Measures

Participants’ gender was assessed as male, female, and another gender. Race and ethnicity were independently assessed and combined as Hispanic and White non-Hispanic and Asian non-Hispanic. Professional characteristics of the participants were assessed to characterize the breadth of training across the sample. Participant degrees, medical specialty, and length of employment at their primary workplace in months were assessed using open response fields. Participants were also asked to indicate which work environments they were employed in, with response options including: academic institution; ambulatory surgery center; free standing clinic; health department; hospital; substance use treatment center; harm reduction organization; something else.

A semi-structured interview guide reviewed for content by substance use epidemiologists, a physician, and a research director of a syringe services program was used to facilitate interviews. The guide explored: (1) providers’ patient population; (2) treating xylazine health effects, with subsections for xylazine testing, overdose, skin wounds, withdrawal, and MOUD; and (3) reflections on organizational capacity to manage xylazine-associated health effects. The guide also included probing questions as needed.

### 2.2. Analysis

Interviews were transcribed verbatim with the support of Rev. (an online, secure, transcription platform), reviewed and deidentified by the first author. Transcripts were analyzed following the steps of Rapid Qualitative Analysis (RQA) (Hamilton, 2013). RQA is a five-step, team-based, iterative approach for understanding meaning from qualitative data to efficiently answer a timely research question. Analysis was led by the first author and supported by the second author, a doctoral candidate with expertise in substance use treatment and qualitative research. In step one, the two analysts mapped the interview guide questions to thematic areas (i.e., domains) (See Appendix A). In step two, the analysts created a summary template to document domain summaries and exemplary quotes for each domain. In step three, the analysts independently tested the summary template on transcript one and were attentive to whether data mapped to the domains and domain names were clear and comprehensive. Following this test and discussion, some domains were reordered, renamed, and divided into subdomains to achieve mutual compatibility with the template. For the fourth step, the remaining 11 transcripts were summarized independently by both analysts. The analysts then convened to identify inconsistencies in domain summaries and to generate a reconciled summary template per transcript. Afterwards, for the fifth step, the first author utilized a spread-sheet matrix to organize domain summaries from the reconciled summary templates by participant. The matrix allowed the first author to identify commonalities and differences across transcripts within each domain that informed narrative summaries that are the basis of the results. The second analyst verified summaries. All team members, with expertise spanning substance use and clinical medicine, reviewed and interpreted themes collaboratively.

## 3. Results

Eight physicians, two nurse practitioners, one physician associate, and one nurse participated in this study. Eight participants were women, three were men, and one was gender fluid. Most (*n* = 10) were White non-Hispanic, one was Asian non-Hispanic, and one was Hispanic. Eight participants were board-certified physicians across addiction (*n* = 7), internal medicine (*n* = 4), infectious disease (*n* = 3), and emergency medicine (*n* = 1) specialties. Participants worked in academic institutions (n = 7), free-standing clinics (*n* = 5), harm reduction organizations (n = 4), substance use treatment programs (n = 4), hospitals (*n* = 2), and a federally qualified health center (n = 1). Participant length of time employed at their current workplace ranged from 2 to 168 months (average of 49.75 months, about 4 years).

Interview duration ranged from 28 to 65 min. The RQA produced five themes regarding healthcare providers’ emergent xylazine treatment practices: (1) xylazine exposure is inferred by clinical presentation, rarely by toxicology screening; (2) respiratory support and monitoring are essential when responding to xylazine-involved overdose; (3) wound chronicity, limited treatment guidelines, homelessness, and stigma complicate xylazine-associated skin wounds healing; (4) withdrawal from xylazine dependence typically co-occurs with opioid withdrawal; and (5) xylazine may be worsening experiences of precipitated withdrawal when inducting onto buprenorphine. Themes and additional exemplary quotes are presented in Table 1.

### 3.1. Xylazine exposure is inferred by clinical presentation, rarely by toxicology screening

Participants identified community-based drug checking and toxicology screening as useful tools for xylazine surveillance, though their perceptions of relevance to clinical care management differed. Participants noted that community-based drug checking (e.g., xylazine test strips, Fourier-transform infrared spectroscopy) results can help patients make decisions about safer-use practices and help providers counsel on harm reduction. At the same time, participants acknowledged that toxicology screening for xylazine can objectively verify exposure and inform population-level drug supply surveillance efforts. However, participants explained that toxicology results, if ordered at all, rarely alter clinical management of xylazine exposure. Rather than rely on screening, participants noted that xylazine exposure can usually be inferred from knowledge of the local drug supply and from characteristic clinical manifestations of xylazine exposure like prolonged sedation, skin wounds, and difficult-to-treat opioid withdrawal. A Massachusetts addiction and internal medicine physician stated:

If we can diagnose something clinically, why do I need to send off any diagnostic test? I think that xylazine point of care testing has a much more important role in terms of harm reduction, overdose prevention, and wound prevention than it does in terms of addressing the consequences of xylazine, which are overdose and wounds.

Participants also stated that practical challenges complicate testing for xylazine in clinical settings, reinforcing the reliance on clinical assessments to presume exposure. Participants described a lack of access to a xylazine immune assay screen for point-of-care testing, and when a test is ordered, results come in after a patient has left care. Additionally, as articulated by a physician, xylazine’s short half-life (i.e., 23–50 min as reported in Papudesi et al., 2025) and the lag between the start of a patient’s visit and when an addiction medicine consult might order comprehensive testing narrows the detection window. These logistical challenges undermine the utility of testing and potentially result in false negatives: “*I will say that if it* [xylazine testing] *is obtained, often it’s delayed. And my understanding is that the detection tends to be a relatively short window”* (Rhode Island Physician, Addiction and Internal Medicine).

Although toxicological testing for xylazine typically lacks clinical utility, some participants noted that this could be useful given that limited drug surveillance obscures xylazine’s true prevalence in the drug supply. Indeed, participants’ estimates of xylazine exposure among patients ranged, with some participants estimating as few as 1 in 10 patients while others estimated as many as 9 in 10 patients using fentanyl are exposed. Moreover, participants noted that enhanced screening could also inform harm reduction messaging about safer use practices and detect other emergent adulterants. For example, Pennsylvanian participants who were contending with the rapid emergence of medetomidine, a veterinary sedative stronger than xylazine, in their local opioid supply at the time of study participation especially underscored the need for comprehensive drug supply testing.

### 3.2. Respiratory support and monitoring are essential when responding to xylazine-involved overdose

Participants emphasized that xylazine’s presence in the unregulated fentanyl supply has changed how overdose presents and is reversed. One New England participant anecdotally noted code times (i.e., the time spent providing critical life-supporting care) for opioid-related overdose events have nearly doubled in the xylazine era. Naloxone administration is necessary to address opioid contribution to overdose but insufficient to attenuate xylazine’s contribution to overdose, meaning patients often remain sedated with low respiration rates after naloxone administration. Thus, participants emphasized the importance of respiratory support and extended monitoring to address respiratory depression and sedation driven by xylazine. Some participants noted that those unfamiliar with xylazine’s sedative property, particularly first responders, may repeatedly administer naloxone, believing that the patient has consumed high dose opioids. Repeated naloxone administration can cause precipitated withdrawal which, as one New England participant noted, can cloud the “*clinical picture*” of a patient’s health:

*If I’m slamming people with Narcan, I’m just going to complicate that clinical picture. When they wake up and they’re vomiting, I don’t know if they’re vomiting, they had a significant head strike or they’ve got the flu or something like that. It’s hard to differentiate. So, if we can give Naloxone without inducing withdrawal, it uncomplicates the picture for us*. (Massachusetts Nurse Practitioner, Addiction Medicine)

While all participants emphasized the importance of administering naloxone to respond to suspected opioid-involved overdose, participants advocated for compassionate naloxone use in controlled medical settings with certified medical personnel when xylazine involvement in overdose is suspected. Compassionate naloxone use involves monitoring and “oxygen first” protocols with naloxone titrated to the needs of the patient. Participants employed within harm reduction organizations in New England and Pennsylvania have built respites for patients to wait out sedation under the monitoring of trained safety personnel ready to administer oxygen and naloxone if needed. One internal and addiction medicine physician who supports a New England-based overdose prevention center shared:

It’s compassionate Narcan. So, you don’t give Narcan until your respiratory rate is four because you can support someone with just oxygen instead of giving them Narcan, sending them in severe withdrawal, and then they go out and use and overdose again.

In these spaces, patients can be safely positioned to maintain air-ways, oxygen levels can be monitored with pulse oximeters, and supplemental oxygen and naloxone can be administered as needed. Respites also protect patients from sexual assault, theft, and injury that accompanies xylazine sedation on the street, which participants shared is an unfortunately common occurrence: “*a common anecdote I’ve heard from patients talking about one of the things they hate most about xylazine is that they’re so sedated, they’re unable to protect their own belongings*” (Rhode Island Physician, Addiction Medicine).

### 3.3. Wound chronicity, limited treatment guidelines, homelessness, and stigma complicate xylazine-associated skin wounds healing

Participants explained that xylazine wounds are heterogeneous in appearance and size, and typically manifest as small pimples or blisters, most often on extremities. Several participants noted that xylazine wounds differ from injection-related abscesses by their chronicity and the potential for extensive necrotic tissue with eschar and progression into large wounds that may reach bone. Some advanced wounds may warrant surgical intervention. While xylazine wounds typically appear around injection sites, some participants recollected patients who only smoke or snort xylazine-adulterated fentanyl who developed wounds on extremities and nasal passages. Participants anecdotally speculated that patients using opioids who also use stimulants and skin pick may predispose themselves to wound development, which participants believed complicates healing by scab picking.

Now several years into xylazine’s presence in the drug supply, participants described greater confidence in how to treat xylazine wounds to prevent advanced progression. Nonetheless, treatment approaches are heterogeneous because there are not widely adopted xylazine wound care guidelines. Participants stressed that most xylazine wounds can be managed in outpatient settings and adequately heal if properly cared for. Participants who worked at harm reduction organizations adapted to the clinical needs of patients, hiring wound care nurses and stocking wound care supplies when xylazine emerged. A Massachusetts-based addiction and internal medicine physician shared:

The first place I’ve always started is referring them to a place that cares for people who use drugs and have a lot of expertise with people who use drugs because those people have been forced to become experts in xylazine wounds and they’re now extremely good.

Aside from harm reduction organizations with dedicated wound care services, participants noted that many outpatient settings lack the adequate wound care supplies and knowledge needed to properly treat xylazine wounds. Moreover, participants emphasized that unaddressed behavioral and environmental factors often prohibit wound healing, leading to life threatening infection, amputation and physical disability. Infection is of particular concern for unhoused patients who may lack access to hygienic living environments, clean water, and storage for wound equipment:

*For my patients who are experiencing homelessness or unstable housing situations and who already have many structural barriers to protecting their own health and accessing care, having additional wounds that can impede things even further*. (Rhode Island Physician, Addiction and Infectious Disease)

In one instance, a participant recounted patients who cannot use their arms because of severe xylazine wounds, making independently changing dressings an impractical ask. All participants conveyed that patients feel shame about their wounds, which can delay or prevent help seeking, as articulated by a Pennsylvania-based infectious disease doctor working in a harm reduction organization:

I have a lot of patients who I’ve known for three years now, and it takes a lot for them to let me change their wounds. They’re very embarrassed by it. They’re embarrassed by the smell; they’re embarrassed by the appearance. And so, I think having them trust a medical provider to do their wound care takes a lot.

Given the potential complexity of xylazine-associated wounds, participants believed that interdisciplinary collaboration between nurses and physicians with expertise in addiction, infectious disease, and surgery is needed when creating treatment plans for advanced wounds. Collaborative care models are not without challenges, as participants noted that some surgeons may be reluctant or refuse surgery on patients who continue to use drugs, and others may provide unnecessarily aggressive debridement. For patients with damaged veins, wounds can become an injection site for continued use which harms the wound bed. Participants noted that continued drug use despite wound development, particularly when patients inject into their wounds, has worsened what was already a tenuous perception of people who inject drugs by healthcare providers:

*They [patients] know if they go in and say, “Hey, I injected and here’s what happened, or I was just injecting and suddenly this wound got bigger and bigger, help me.” People are going to look at them and have stigma against it. “Oh, you did it to yourself. You’re not worthy of care because you’re an addict.”* (Rhode Island Physician, Addiction and Internal Medicine)

### 3.4. Withdrawal from xylazine dependence typically co-occurs with opioid withdrawal

Participants concluded that it is challenging to determine whether xylazine dependence is associated with a distinct withdrawal syndrome because xylazine and fentanyl are typically consumed together along with other drugs. Thus, most participants characterized xylazine withdrawal as complex, with symptoms of alpha agonist withdrawal like pronounced anxiety, restlessness, and agitation that exceeds what is typically observed during opioid withdrawal. Participants working in outpatient settings were much less familiar with xylazine withdrawal, having rarely encountered this phenomenon because most patients in the outpatient setting are not observed when in withdrawal. Conversely, participants working within the hospital setting were more likely to encounter patients in withdrawal because of lengthy hospital stays.

Participants stressed that patient-reported experiences of withdrawal, along with an understanding of whether xylazine is in a patient’s drug market, should inform care plans. One Pennsylvanian addiction and infectious disease physician shared:

The literature to date has been sort of hemming and hawing whether or not our patients have had a distinct xylazine withdrawal syndrome. I think that I have to trust that my patients are the experts, and while there may not be literature that suggests that there is a definite xylazine withdrawal syndrome, these are the clinical manifestations and we have to treat their symptoms.

Generally, participants’ treatment of withdrawal in patients using xylazine-fentanyl combinations has built on opioid withdrawal management protocols to include clonidine as treatment for xylazine-associated agitation and anxiety and consideration of buprenorphine induction. Ultimately, participants approach management of xylazine-associated withdrawal on a case-by-case basis using a variety of medications, and as such, advocated for clearer clinical guidance in how to manage this syndrome. Clear guidance is needed because inadequately managed withdrawal in hospital settings can lead patients to self-directed discharge, high-dose use, and compromise receipt of treatment for problems like xylazine-associated skin wounds.

### 3.5. Xylazine may be worsening experiences of precipitated withdrawal when inducting onto buprenorphine

Participants affirmed that the introduction of xylazine into the fentanyl supply has complicated induction onto MOUD. In knowing that buprenorphine induction typically begins after abstaining from opioid use to avoid precipitated opioid withdrawal, participants were asked how well patients tolerate induction in the era of xylazine. Participants reported that patients using xylazine adulterated fentanyl may struggle to initiate buprenorphine, even when abstaining, as they often experience a protracted withdrawal syndrome. A Rhode Island nurse practitioner working within an outpatient addiction treatment clinic shared: “*I do feel like we’re seeing people that have that sort of protracted* [withdrawal] *where they sort of feel crappy for days as you’re trying to induct them* [onto buprenorphine]”. Relatedly, participants noted a key challenge with buprenorphine induction is that xylazine has made it complicated to accurately assess whether a patient is in moderate opioid withdrawal given how xylazine has changed experiences of withdrawal. In some cases, participants reported that this misread of withdrawal has led prescribers to believe that buprenorphine induction can begin with minimal risk of precipitated withdrawal – an error that triggers precipitated withdrawal. Addiction physicians were unsure what may drive this protracted withdrawal. Participants presume that poor experiences with buprenorphine induction, driven by xylazine, is fueling a growing disdain for buprenorphine among patients. In response to induction difficulties, participants have turned to three other approaches for patients seeking MOUD: 1) microinduction of buprenorphine, 2) macroinduction of buprenorphine, and 3) methadone.

Participants working in both inpatient and outpatient settings have titrated patients up from small doses of buprenorphine (on average 0.5 mg from a 2 mg film) while the patient titrates down use of unregulated fentanyl and utilizes comfort medications for opioid withdrawal. One nurse at a Rhode Island opioid treatment program described:

They can still use, so it’s like a formula. You start with a fourth of a strip, have them continue to use all the way to day seven, and you gradually increase. And then the idea is that they don’t use and then they get a full thing. So, you’re not putting them in a precipitated withdrawal.

Microinductions may be easiest in inpatient settings and more challenging for some patients in outpatient settings. However, limited hospital beds and financial resources to cover the length of care can make microinductions challenging to complete while hospitalized. For patients who cannot tolerate microinduction because of protracted withdrawal, or for patients disinterested in microinduction, a few participants reported comfort and success with macroinduction buprenorphine in controlled settings. Described as “flooding” a patient’s receptors to override the presence of fentanyl and xylazine, macroinduction can be effective but not always well-tolerated by patients:

*We switch to the high dose or the macro if somebody does go into precipitated withdrawal…because the buprenorphine binds so much tighter than everything you get complete receptor saturation. It is kind of amazing to see folks go from precipitated withdrawal to feeling* – *it’s not that they feel like back to a hundred percent immediately, but a pretty tangible difference pretty quickly if you get the doses high enough.* (Rhode Island Physician, Addiction and Internal Medicine)

Participants comfortable with macroinduction anecdotally remarked that many prescribers are reluctant to utilize the high-dose buprenorphine approach when inducting patients. Some Pennsylvania participants described the innovative use of monthly long-acting injectable buprenorphine for rapid induction with success for patients dependent on xylazine-fentanyl combinations. Although methadone is typically preferred by patients because it is a full agonist and avoids the risk of precipitated withdrawal, some participants described barriers to accessing methadone. Specifically, participants noted that patients who are unhoused and for whom the sedative effects of xylazine make it challenging to appear for daily dosing at an opioid treatment program face difficulty adhering to methadone. Lastly, Pennsylvania participants noted that the supplanting of xylazine with medetomidine appeared to be associated with a withdrawal syndrome more intense than xylazine.

## 4. Discussion

This qualitative study of physicians and other healthcare providers with experience managing the effects of xylazine produced novel and timely insights into the clinical management of xylazine. Though toxicological testing for xylazine would help to produce a robust understanding of who is exposed to the adulterant, participants concluded that testing is often not necessary, as exposure can be inferred based on clinical presentations. As previously documented (Zagorski et al., 2023), xylazine-involved opioid overdose requires a nuanced response, with participants noting that respiratory support is central to addressing respiratory depression. These results build on this understanding by showing that, in controlled settings with trained personnel such as in respite programs or overdose prevention centers, respiratory support with monitoring and naloxone titration can safely be prioritized over repeat naloxone doses to minimize risk of precipitated withdrawal. Consistent with extant literature (Sue & Hawk, 2024), xylazine-associated wounds are the most visible indicator of xylazine exposure that also cause patients shame. While these wounds can progress to become debilitating and infected, participants stressed that xylazine-associated wounds can largely be treated in outpatient settings equipped with trained clinicians and supplies to prevent progression. Findings emphasize that xylazine-associated withdrawal is a poorly understood clinical syndrome and advance our understanding of treating this phenomenon by illustrating how providers have adopted a multimodal approach to treatment of xylazine withdrawal; micro and macro buprenorphine induction in tandem with adjunctive therapies like clonidine and comfort medications hold promise in helping to relieve some withdrawal symptoms. Collectively, findings indicate that the clinical effects of xylazine do not occur in isolation from other substances and intensify one another, demonstrating the increasingly complex harms of the adulterated, volatile drug supply and underscoring the need for integrated care for PWUD.

In this study, participants reported rarely testing for xylazine exposure in clinical settings, primarily because exposure can be inferred based on clinical presentations. Diagnostic assays for xylazine are not readily available in clinical settings (D’Orazio et al., 2023), reinforcing reliance on the presumption of exposure based on clinical presentation. The lack of robust xylazine exposure monitoring through testing explains the variation in participants’ estimates of their patient population exposed to xylazine (10–90%), which aligns with other work that reported variable provider estimates of xylazine exposure due to limited standardized testing (Hill et al., 2024). While participants noted that community drug checking programs can help inform patients about safer use strategies, these results seldomly impact clinical management because drug checking in the community occurs outside of the clinical encounter. Nonetheless, strengthening drug checking services is an increasingly important aspect of harm reduction programming to help people make informed decisions about their use. To that end, there remains a need to improve the accuracy of xylazine test strips before this harm reduction strategy can be deemed fully reliable (Thompson et al., 2024). We also found that while participants were largely confident in their ability to recognize the signs of xylazine exposure, it was noted how limited to no toxicology testing obscures an epidemiologic understanding of exposure to the adulterant; this reinforces a reliance on imprecise measures, including drug seizure, community drug checking, and forensic data. Integrating information across these data sources would enable timely and accurate provider-focused public health communications. Moreover, robust monitoring systems would reduce the odds that clinicians first learn of xylazine from a patient interaction, as was found in this research and elsewhere (Reed et al., 2024). Quickening the pace at which the analytes of standard urine drug screens can be updated to account for emergent adulterants in the unregulated drug supply is also an important area of future research in the present era of drug market volatility. For example, medetomidine, a strong veterinary sedative that is supplanting xylazine in some drug markets (Sibley et al., 2025), is just one contemporary example of a drug market shift that addiction medicine struggles to keep pace with.

Our findings indicate that while naloxone should still be administered in the scenario of suspected opioid involved overdose, xylazine has necessitated more nuanced approaches to naloxone dosing and heightened the importance of respiratory support. This finding converges with other research with PWUD exposed to xylazine, healthcare providers, and harm reduction workers indicating that strategies in addition to naloxone, such as rescue breaths, are needed to support people experiencing xylazine-involved overdose (Eger et al., 2024; Quijano et al., 2023). The extent to which the importance of rescue breaths in responding to xylazine-involved overdoses has been integrated into Overdose Education and Naloxone Distribution trainings and adopted by PWUD is not fully understood and is a direction for future research. Qualitative findings from Philadelphia indicate that PWUD understood the importance of rescue breaths (Reed et al., 2025), whereas other Maryland-based qualitative work found that PWUD are not regularly providing respiratory support when responding to overdose (Sisson et al., 2025). Thus, there is a need to widely communicate about and measure the effectiveness of modified overdose response protocols in the xylazine era.

Participants recognized that xylazine has changed the physiology of how overdoses involving opioids and xylazine occur and some called for the adoption of compassionate approaches to naloxone use. Extant work has documented the discomfort and acute withdrawal experienced by some PWUD dosed with intranasal naloxone (Reed et al., 2025). The participants of the present study have seen this phenomenon worsen because of a proclivity to repeatedly administer naloxone in excess by those unfamiliar with xylazine. Though often necessary to address opioid contribution to overdose, sudden withdrawal caused by excessive naloxone dosage is associated with opioid use and overdose upon arousal (Kahn et al., 2022). Moreover, as our participants noted, precipitated withdrawal muddies the clinical picture of a patient's health upon revival. We found that harm reduction organizations, and particularly overdose prevention centers, have crafted and implemented compassionate naloxone protocols that center on patient safety. These efforts should be scaled. Organizations led by and who serve PWUD are best positioned to refine compassionate naloxone approaches and should play a central role in designing training efforts for first responders and other healthcare personnel in the conduct of this measured approach to naloxone dosing that prioritizes respiratory support and patient safety.

Findings from this study underscored that without early intervention, managing xylazine-associated wounds requires substantial resources and coordination, and stigma significantly undermines timely care. Participants especially noted that wound care needs are heightened for patients with unstable housing who lack access to consistently hygienic environments as has been noted by others (Carroll, 2024). With early intervention and consistently changed dressings, participants stated that xylazine-associated wounds can heal well and be prevented from progressing to have serious and potentially limb and/or lifethreatening complications. Participants called for standardized wound care management protocols, such as the one published by Shang et al., 2025, which was published while data collection for the present study concluded. Adding to the complexity of wound management is that many patients co-use stimulants in addition to xylazine-fentanyl combinations; how polysubstance use patterns affect xylazine-associated wound development and management remains to be understood. Also foregrounded was the intensity of the shame people with xylazine-associated wounds contend with that even highly trusted providers struggle to help patients process, as has been reported previously (Kelly et al., 2025). Our findings suggest that addiction medicine providers are having to advocate even more strongly for patients, address stigma within the medical community, and do so while having limited evidence-based protocols to address patients’ drug use that reinforces wound development. The present findings add nuance to our understanding of stigma toward xylazine-associated wounds by showing how some surgeons endorse the sentiment that PWUD with xylazine-associated wounds have harmed themselves; other work has found that stigmatizing beliefs have led some patients to delay or avoid getting needed care in regions of the US where xylazine is prevalent (Kelly et al., 2025). Street medicine organizations that bring supplies and clinicians to people where they reside can help to reach patients with wounds who may avoid clinical settings to protect themselves from stigma (Rasul et al., 2023).

Participants in this study believed xylazine dependence is possible, and this has complicated MOUD initiation, requiring what participants characterized as individualized, flexible, and multimodal treatment options. Aside from subjective experiences of anxiety, restlessness, and agitation, participants did not note objective indicators (e.g., hypertension) of xylazine withdrawal, consistent with a hospital-based cohort study of xylazine-exposed patients (Thakrar et al., 2025). What participants centered as most critical is listening to patients’ characterizations of withdrawal to inform conversations about initiation of MOUD. Some participants reported successful use of clonidine in helping to address xylazine withdrawal. Micro- and macroinduction onto buprenorphine are two initiation approaches that, according to anecdotal evidence from the present study, may help avoid precipitated withdrawal and achieve stability depending on the induction context, patient preferences, and provider comfort level. Emergent cohort research from Philadelphia has shown some initial promise of microinduction of buprenorphine in combination with mu-agonists and other supportive medications (London et al., 2024). Methadone was regarded by participants as effective treatment for xylazine associated withdrawal, though methadone alone does not adequately address xylazine’s contribution to the withdrawal syndrome without adjunctive medications. Moreover, participants noted that long standing barriers to accessing and receiving methadone continue to prevent its widescale adoption, which is well documented in the literature (Cernasev et al., 2021). Perhaps of all xylazine-associated health effects, the need to establish effective xylazine-associated withdrawal treatment is paramount because this study indicated that untreated or undermanaged withdrawal is associated with avoidance of care and reinforced drug use.

There are some limitations to this research. Like all qualitative research using convenience samples, findings are specific to the clinical perspective and practices of the participants and may not transfer to professions not represented in this study. However, findings are transferable to drug markets in which xylazine is prevalent and may have the greatest utility in drug markets where xylazine is just beginning to emerge. The quickly changing drug market and xylazine knowledge landscape evolved during data collection, but we were still able to assess emergent drug supply changes, such as medetomidine. Rapid qualitative analysis prioritizes summarization to gain an understanding of a particular topic, which can minimize meaningful nuance in data that other qualitative approaches afford. Because this study relied on self-reports to determine appropriateness to participate in an interview about clinically managing xylazine exposure, there was an array of experience managing xylazine exposure within the sample, resulting in some providers having greater knowledge and familiarity than others.

## 5. Conclusion

Findings from this study show that the clinical effects of xylazine do not occur in isolation and instead amplify one another. Pain and shame associated with xylazine wounds can reinforce continued drug use, which in turn increases overdose risk and can accelerate wound progression, particularly for those who inject into wound beds. Ongoing drug use also increases the risk of dependency and withdrawal, while the fear of experiencing withdrawal in treatment settings can delay careseeking, worsening clinical outcomes related to wounds and MOUD induction. Future work should develop and evaluate training interventions for providers working across care settings, with a focus on stigma reduction and improving competency in recognizing and treating xylazine’s effects. Creative efforts to modernize drug monitoring systems to account for the emergence of new adulterants, investment in stigma reduction efforts, and testing of MOUD induction protocols are needed.

## Figures and Tables

**Table 1:** Exemplary quotes by theme.

Theme 1: Xylazine exposure is inferred by clinical presentation, rarely by toxicology screening	*I will say part of the reason I think for that is* *the test takes longer to come back and so it’s* *not necessarily going to guide your acute management… I would not necessarily expect an emergency physician to order it because they’re like, potentially this patient is going to be leaving by the time this results or they’re being admitted. And I think it’s not front of mind necessarily*. – Rhode Island Physician, Addiction and Internal Medicine *Really some issues in timeframe turnaround with the laboratories that we were sending it to, and we would rather know in real time what’s in somebody’s urine tox screen than finding out two weeks later what was in it two weeks ago… It’s almost like we see it so frequently now it’s more commonplace that I just assume their supply is going to have xylazine rather than probably that it doesn’t.* – Rhode Island Nurse Practitioner, Addiction and Family Medicine *I don’t know if there’s a lot of added value of sending out an expensive test that takes one to two weeks to come back. And at least in the acute care setting where I work, that patient may be discharged. So, I think it’s just like any clinical thing. What is the value of the diagnostic test? How is it going to help you move forward and give the appropriate treatment?* - Massachusetts Physician, Addiction and Internal Medicine *I admit that early on I was excited about giving out xylazine test strips. Then I heard a lot of information about false positives and that they weren’t very accurate. Someone recently told me that that accuracy has dramatically improved, which I was excited to hear, but I think not to the point where there was a newfound enthusiasm or eagerness to try to obtain those. So I think accessibility and questions about their accuracy have been the biggest barrier to providing xylazine test strips in the outpatient recovery clinic.* - Rhode Island Physician, Addiction Medicine *We are mandated by the commonwealth of Pennsylvania to report fentanyl positive urine toxicology screens. They’ve asked us to do xylazine. That is theoretically, I still believe, a mandate. The caveat to that is understandably, if we don’t have the ability to provide conventional xylazine testing, we don’t have to provide it because again, it doesn’t exist. So functionally there is an unfunded unsatiated mandate that is purely, again, leaving a hole in our public health understanding because we don’t have the technology to facilitate it.* - Pennsylvania Physician, Emergency Medicine
Theme 2: Respiratory support and monitoring are essential when responding to xylazine-involved overdose	*I’ve had a lot of patients talk about giving* *naloxone to someone. I don’t know that I’ve* *ever heard of one of my patients giving* *rescue breaths to someone and that’s probably just a gap in education or implementation of response. But I don’t know of anyone who’s done that, but lots of naloxone giving.* – Rhode Island Physician, Addiction Medicine *A lot of times folks will require supplemental oxygen and additional respiratory and monitoring support that wouldn’t necessarily* (*continued on next page*) *be required if it was a straight opioid overdose. So that certainly complicates the response, particularly in the community setting where there’s less resources available potentially. It’s something that I think the community of people using drugs often has a lot more experience and expertise in monitoring those sorts of overdose events and knowing what to look for and how to respond oftentimes than providers sometimes do. It’s more of a newer phenomenon that we’re learning about.* – Rhode Island Physician, Addiction Medicine and Infectious Disease *I could see EMS or someone who isn’t as familiar with the xylazine side of it giving repetitive naloxone, which then when the person does come to makes them completely miserable and angry. It is definitely challenging because I think your first instinct is giving them more naloxone cause you’re nervous that they’re not waking up and that they’re still like very risk for overdosing, but it doesn’t do anything.* – Pennsylvania Physician, Addiction Medicine, Internal Medicine, Infectious Disease
Theme 3: Wound chronicity, limited treatment guidelines, homelessness, and stigma complicate xylazine-associated skin wounds healing	*Even in my non-IV drug users, IV drug users, we’re thinking about skin wounds. We’re* *thinking about skin wounds at the injection* *site, but even people primarily snorting or primarily snorting or smoking, some have had xylazine skin wounds.* – Rhode Island Physician, Addiction and Internal Medicine *People seem really afraid of it [xylazine associated wounds] and it seems like they’re very hesitant to believe that that would be from xylazine.* – Rhode Island Nurse Practitioner, Addiction and Family Medicine *It’s definitely worse for those who are unhoused. And that is purely from a practical wound care perspective. If someone doesn’t have a stable environment, it’s hard for them to change their wounds or keep wound care supplies. And then obviously in the extreme temperatures when it’s cold outside, it’s very painful to change their wounds. And when it’s super hot, it’s more prone to getting maggots. And so, anyone who’s outside for long period of time, it’s always worse.* – Pennsylvania Physician, Addiction Medicine, Internal Medicine, Infectious Disease *We’ve had some negative interactions with some surgeons sort of being like, we’re not going to provide surgical management of this until this person has X number of months of abstinence and which I think can be, it’s a really difficult thing for the patient to hear and also for us to be - it almost feels like we’re liaising between the patient and another specialty and trying to advocate. I feel like it can be challenging to create a healthcare experience for people that doesn’t feel negative.* – Rhode Island Physician, Addiction and Internal Medicine *I think long-term, being able to have someone in a stable enough environment so that they can heal or having a surgeon willing to do a skin graft or debridement is another problem. I’ve had patients with exposed bones, exposed tendons and no surgeon will touch them. And so getting them* *stable and in recovery long enough despite the pain of having an exposed bone is really hard to get them to the point of even having surgery. And so if they can’t heal on their own, getting that next level of care is really hard to get, especially by surgeons.* – Pennsylvania Physician, Addiction Medicine, Internal Medicine, Infectious Disease *There are structural barriers in terms of accessing additional services, getting to said wound clinic, purchasing the wound care supplies, that sort of thing. There’s also a lot of perceived stigma on the patient side that a lot of folks have unfortunately had really terrible and traumatic experiences in the medical system previously and thus are understandably less excited about seeking care, particularly by a new provider who they may not know or trust.* – Rhode Island Physician, Addiction Medicine and Infectious Disease *I think the challenge in the hospital and outside the hospital is how long these wounds are taking to heal and the challenge of active substance use when trying to recover from these wounds because lots of folks are using the same place to inject, and that really, really impacts the wound’s ability to heal. So, I think that xylazine wounds themselves are horrific. They take a long time to heal, and that’s made more complicated in the context of really active chaotic substance use, which is made more complicated when people don’t have the things to meet their survival needs, like warm, dry clothes, showers, shelter.* – Massachusetts Physician, Addiction and Internal Medicine *There’s this sort of confusion in diagnosis whether or not this is a skin and soft tissue infection, whether or not this is necrotic wound or burn or if this is a necrotizing skin and soft tissue infection. Those are distinct clinical entities that are managed differently. So that diagnosis is something that can be a point of confusion for clinicians that are not addiction trained and or not aware of the changing drug supply and how it is clinically changing people’s symptoms. The second thing is people who use substances are often worried about coming to seek medical care for their wounds because of the healthcare stigma that they faced. And so we often don’t tend to see patients with xylazine associated wounds until they’re much more advanced in stage.* – Pennsylvania Physician, Addiction Medicine and Infectious Disease
Theme 4: Withdrawal from xylazine dependence typically co-occurs with opioid withdrawal	*It [xylazine] tends to prolong the experience* *of the opioid withdrawal.* – Rhode Island Physician, Addiction and Internal Medicine *There is this concept of xylazine withdrawal that I admit, I dunno that I’ve seen robustly. I think people probably do feel a little more sick when they’re going through opioid withdrawal in general, but I don’t know that I’ve seen a ton of like, oh, this is slam dunk xylazine withdrawal rather than, this is kind of a complicated opioid withdrawal. For me, it’s difficult to tease that out perhaps cause I’m not seeing them on a continued basis like in an inpatient or ED setting. So in my mind, and maybe this is overly simplistic, but I kind of view it as opioid withdrawal broadly.* – Rhode Island Physician, Addiction Medicine *There’s a little more agitation the way that xylazine hits the receptors,* *pharmacologically, clonidine maybe makes* *a little bit more sense to treat, but there are slight modifications to the receptors that make clonidine less effective. The best* *medication probably for some of the* *xylazine agitation are benzodiazepines, which some people use for opioid withdrawal broadly. But I think a lot of* *providers don’t have a lot of comfort* *prescribing benzodiazepines to someone who* *has a substance use disorder actively.* – Rhode Island Physician, Addiction Medicine *I think is a big problem is if someone is going* *to the hospital, because they have a very* *serious infection or a serious reason to go* *there, they don’t stay long enough to get taken care of. So I’ve had patients go who* *have endocarditis, bloodstream infections,* *something hemoglobins of four where I’m* *like, ‘you have to go to the hospital. I’m really worried you could die if we don’t get you there and stabilize you’ and they can’t stay for more than 24, 48 h cause the* *withdrawal is so horrible. And part of that is* *there’s a lot of trauma of being in the* *hospital system from prior stuff, but a lot of it is withdrawal and we do a really crappy job of treating it. If they’re in the waiting room for 12 h, that’s 12 h that they’re in* *withdrawal before they even get back and* *get a dose of methadone. And then the* *methadone doesn’t even help the tranq* *[xylazine] withdrawal. It’s hard to keep* *them in the hospital and it’s hard to educate* *the providers about proper ways to manage* *xylazine withdrawal.* – Pennsylvania Physician, Addiction Medicine, Internal Medicine, Infectious Disease *It can be tricky to delineate xylazine withdrawal versus some other substances,* *particularly other sedating substances such* *as benzodiazepines or something, for example, in my experience with xylazine and other alpha adrenergic agents will cause a lot more variability in blood pressure and* *vital signs, which can be often a clue that* *that’s what we’re dealing with in addition to* *the respiratory depression. And those effects* *tend to, they can be prolonged over the* *course of several hours or even longer potentially, depending how much and how* *often the person was using the xylazine.* – Rhode Island Physician, Addiction Medicine and Infectious Disease *When I talk to my colleagues who I would consider other experts in this, what we think* *is that their alpha agonist withdrawal is in many ways very similar to opioid* *withdrawal, hence why clonidine has been* *used for decades as a management tool to* *facilitate relief from symptomatic opioid withdrawal. So I think that a lot of the* *challenges that we were initially trying to* *figure out were that we didn’t really understand how much a separate substance* *that can appear to provide a withdrawal syndrome that looks very similar to what we would commonly call opioid withdrawal,* *how that would impact how we would treat something like that.* – Pennsylvania Physician, Emergency Medicine
Theme 5: Xylazine may be worsening experiences of precipitated withdrawal when inducting onto buprenorphine	*We know xylazine has that protracted* *opioid withdrawal and it’s making people, it* *gives fentanyl legs, but it also gives opioid* *withdrawal legs. And these people are stopping their fentanyl 72 h taking that first dose of Suboxone outpatient or on the phone and then still going into precipitated withdrawal. So that gives us an idea that xylazine might be in their supply because it’s like hanging around those receptors and hanging out around those opioid receptors even though it’s not an opioid. It’s just like making those fentanyl effects last longer. So the traditional, ‘hey, wait a few days and be in severe withdrawal,’ get someone on Suboxone is sometimes not working for people who are heavy xylazine users because it’s sticking around and making it more difficult to get on Suboxone outpatient.* – Rhode Island Physician, Addiction and Internal Medicine *I do feel like we’re seeing people that have that sort of protracted [*withdrawal*] where they sort of feel crappy for days as you’re trying to induct them. And that’s sort of even whether they’re taking the slower path onto buprenorphine for their own sake or whether they want to do it on a quicker scale, you know what I mean, with higher doses of buprenorphine upfront.* – Rhode Island Nurse Practitioner, Addiction and Family Medicine *Our typical management for xylazine withdrawal is clonidine and gabapentin. The clonidine is a part of our order set for the COWS score. There’s an automatic, I tend to, instead of making it per the score, I’ll just make it standing and we’ll have hold parameters for if the patient’s heart rate or blood pressure are too low. But if they’re tolerating it and still having symptoms, then we just increase the dose of the say from 0.13 times a day to 0.23 times a day without it having to be triggered by the COWS.* – Rhode Island Physician, Addiction and Internal Medicine *I give them clonidine because I assume that the supply may have xylazine in it. And if they’re used to like if xylazine is an alpha-2 agonist, I try to substitute that out and try to minimize or mitigate what risk they may have of having some withdrawal from xylazine by stepping that over to clonidine even at least if it’s 0.1. I don’t feel comfortable necessarily just giving them 0.2. I don’t want to tank their blood pressure either, but certainly giving them clonidine that they can take at least twice a day 0.1, I will do that sometimes. I’ve done Gabapentin as well.* – Rhode Island Nurse Practitioner, Addiction and Family Medicine *So for example, if I’m microdosing someone, we do a ton of low threshold buprenorphine we used to kind of have just a discussion around, do you need clonidine as part of withdrawal management? But now it’s just standard. So our literal OBOT protocol includes making sure that you’re giving clonidine as part of the microdosing protocol, and then I go visit people once I get them into the hospital pretty regularly. And so a lot of it is just advocating on behalf of them that I get there and they’re just in extreme anxiety. The problem is getting* (*continued on next page*) *through the emergency room. If people can get through that process and get admitted, they’re really great with giving benzodiazepines anything that’s going to keep people comfortable while they’re there.* – Rhode Island Physician, Addiction Medicine

## Appendix

**Appendix A.** Interview guide questions and probes mapped to rapid qualitative analysis domains

Parent question	Probes	Domain
Could you tell me a bit about your workplace and your role there?	• How would you describe the population of patients you generally see? • What are the biggest barriers to caring for your patient population?	Job Description
When did you start to notice that xylazine might be affecting the patients that you serve?	• Approximately what share of your patient population do you suspect is affected by xylazine? (probe for: what drugs xylazine is typically in) • Which patients are more likely to be exposed/use xylazine than others? (probe for: people using fentanyl, people using stims) • What is your sense about whether people are seeking out xylazine or trying to avoid using it?	Xylazine emergence and prevalence
What characteristics are suggestive of xylazine’s potential involvement in patients’ health problems?	• In what ways, if at all, do these characteristics differ across populations? (probe for: race, ethnicity, age, gender, opioid vs stimulants vs polysubstance use, etc) • How can you differentiate xylazine’s health effects from the health effects of other adulterants in the drug supply?	Xylazine hallmarks
How does your organization test for xylazine? (probe for in-house testing, out sourced testing)	What are some reasons why providers might seek out confirmatory testing of xylazine exposure? • How do the results of toxicology tests showing the presence of xylazine impact the care you provide for patients?	Xylazine testing
How does overdose look different in the era of xylazine?	• What characteristics, if any, distinguish a xylazine-involved overdose from a non-xylazine involved overdose? • What are current best practices for responding to suspected xylazine-involved overdose?	Xylazine overdose
Please describe a xylazine associated skin wound.	• What distinguishes a xylazine-involved skin wound from a skin wound that does not involve xylazine? • What treatment approaches, if any, do you use for skin wound treatment that you would not recommend if xylazine is involved? • What are some of the challenges you face in managing the complexity of xylazine-involved skin wounds? • How can patients self-manage the treatment of xylazine involved skin wounds? • How have referrals to infectious disease, wound care specialists changed with the rise of xylazine? What about referrals to social service organizations? • What concerns do you have about the long-term management of xylazine-associated skin wounds? (probe for: delayed healing, mental health impact etc) • What do we still need to know about xylazine skin wounds?	Skin wound appearance Skin wound treatment Skin wound long-term impact Skin wound research
To what extent, if any, has xylazine changed how people experience fentanyl withdrawal?	• What differences in withdrawal experiences have you observed among different patient populations? o Probe for: pregnant people, polysubstance use, people with severe wound-related pain, etc. • How, if at all, can xylazine withdrawal be distinguished from withdrawal of other substances? o Is this distinction important? • How do you manage xylazine involved withdrawal? (probe for use of mu agonists) • What has worked well? • What has not worked well? • What are the implications of xylazine involved withdrawal being inadequately treated? Probe for: wound care, self-directed discharge • What do we not yet know about xylazine withdrawal?	Xylazine withdrawal description Xylazine withdrawal management
How do you approach starting medications for opioid use disorder in patients who have been exposed to xylazine?	• What works well? • What challenges have you faced doing this. Are there challenges specific to certain medication types? • What do we still need to know about initiation and adherence to medications for opioid use disorder for patients using xylazine?	MOUD induction
What are the resources like at your institution to provide care for patients exposed to xylazine?	• How confident do you feel in being able to counsel patients about xylazine’s effects? • What strategies would you generally recommend to your patients about staying safe when using xylazine? • To what extent do you feel you are able to help patients manage the stigma of substance use, including the visible nature of xylazine-associated wounds?	Xylazine care resources Xylazine counseling
Think back on the patients you have seen who you believe have been exposed to xylazine. Could you share a case that you felt was particularly complex or nuanced?	• Could you describe a case that influenced your understanding of xylazine and its effects?	Xylazine case
How do you respond when a patient tells you that they have been managing their own medical needs rather than accessing formal care?	• What are reasons why patients are managing their own medical needs?	Self-management
